# Supplementary material for: Mosquitoes cloak their legs to resist insecticides
Source: Proc Biol Sci. 2019 Jul 17;286(1907):20191091. doi: 10.1098/rspb.2019.1091 (PMC6661348; doi:10.1098/rspb.2019.1091)
Supplement: Results and figures [file rspb20191091supp1.pdf]

Electronic supplementary material

Supplementary material Text

1.1 The leg proteome of An gambiae female mosquitoes

Leg proteins (approximately 90 legs per sample, three biological replicates) were extracted from resistant (VK7) and susceptible N'Gusso (NG) *An gambiae* mosquitoes and their polypeptides were separated on SDS-PAGE. Fifteen gel slices were analyzed per sample using a n-LC MS/MS Orbitrap mass spectrometer, as previously described (1). Under stringent criteria (99% confidence of identification in both peptide and protein level), 1120 proteins were identified in the leg, representing 10% coverage of the theoretical gene-encoded whole proteome (UniProt 2018). Clusters of Orthologous Groups of proteins Enrichment analysis by DAVID showed that a variety of house- keeping functions, such as ones involved in posttranslational modifications, protein turn over and chaperones (27 proteins), energy production and conversion (9 proteins), and lipid metabolism (13 proteins) were enriched in the legs (Figure S1A).

1.2 Differences in the leg proteome of insecticide resistant An gambiae mosquitoes compared to susceptible

Protein expression levels were then compared between resistant and susceptible legs. In total 316 proteins were differentially expressed among the two data sets, 156 of them being up-regulated (13.9% of leg proteome) and the rest 160 (14%) showing reduced expression in the resistant legs (Figure S1B). Gene Ontology Enrichment analysis of the differentially regulated proteins, using the g:Profiler bioinformatics software tool revealed two categories, structural constituent of cuticle (20 proteins found with the certain GO term) and hydrogen anion transporter activity (6 proteins) molecular functions to be enriched in the dataset of the over-expressed leg-proteins in the resistant legs (Figure S1C, orange bars). Furthermore KEGG-pathway enrichment analysis of the over-expressed proteins indicated oxidative phosphorylation as the primary pathway, comprising the 6 proteins with hydrogen anion transporter activity (see above) and 3 more proteins annotated to mitochondrial respiratory chain complex (Figure S1C, orange bar). In contrast the majority of the down-regulated proteins in the resistant legs depicted a series of metabolic biological processes (GO_BP: small molecule metabolic process: 32 proteins, IMP metabolic process: 5 proteins, nucleoside monophosphate biosynthetic process: 6 proteins) significantly enriched (Figure S1C, black bars). GO molecular function enrichment

analysis in the same data set, emerged ligase activity (7 proteins), hydroxymethyl-, formyl- and related transferase activity (3 proteins), anion and small molecule binding (31 proteins each), as well as translation initiation factor activity (5 proteins) (Figure S1C, black bars). KEGG-pathway enrichment analysis of the down-regulated proteins in the resistant legs confirmed that the most prevalent pathways were Metabolic pathways (39 proteins), Carbon metabolism (9) Tryptophan metabolism (4 proteins) (Figure S1C, black bars).

Structural constituent of cuticle was the most enriched cluster in the up-regulated pyrethroid resistant leg proteome, compared to susceptible, showing a substantial 5.1 enrichment ratio in the set of the over-expressed proteins (Figure S2A). A detailed heat map in figure S2B shows differential expression levels of all CPs detected in leg proteome.

Moreover, twenty-one proteins- members of families previously associated to detoxification, antioxidants, stress responses and penetration resistance were significantly differentially regulated in the resistant legs (Table S3). In details, two P450's (CYP4C27 and CYP4D17) were found significantly down-regulated in the resistant legs. Two GST's, GSTe3 and GSTmic1 proteins, were significantly over-represented in the resistant legs, while GSTO1 was down regulated (Table S3). Additionally, one superoxide dismutase (CUSOD3) was significantly up-regulated in the resistant legs (Table S3). Two carboxyl esterases identified in the resistant legs, were down-regulated. Finally, out of eight Odorant Binding proteins (OBPs) identified in the legs, two of them, OBP10 and OBP57, were significantly up-regulated, whereas five more OBPs were significantly down-regulated in the resistant legs (Table S3).

1.3 Chitin content of resistant An gambiae female mosquito bodies.

Glucosamine from body extracts (eight female mosquitoes per replicate analyzed and measurements were repeated three times) was measured in both resistant (R - VK7: 1.46 mgr of D-glucosamine/ mgr of mosquito bodies ±0.49) and susceptible mosquitoes (S – NG: 1.12 mgr of D-glucosamine/ mgr of mosquito bodies ±0.16). This difference is not statistically significant, as determined by t-test (non-significant) and the data are presented as means + SEM (Figure S5).

Materials and Methods

1. Random Forest Classification of CHC species

A Random Forest Classification strategy was employed as a means a) to assess the level of discrimination that could be achieved from the CHC profiles and b) to obtain a set of CHC signatures that could in principle define the susceptibility of the insects and the body part, from where the sample was taken. Starting from the complete dataset we used a 70/30% split for training and test sets respectively and built 1000 RF models with 500 trees each, using 10 variables at each split. From the 1000 RF models the one with the lowest error rate was chosen and we obtained the variables with the greatest importance on the basis of the higher Mean Gini Decrease (MGD). By assigning “predictor” status to CHCs with MGD greater or equal to one and a half times the mean (MGD (CHC)>=1.5*mean (MGD)) we selected CHCs that could classify samples with great accuracy. For instance, when applied to the complete dataset and using Susceptibility status as the target variable (i.e. the one to be predicted) we qualified 6 CHCs (corresponding to peaks 24, 15, 19, 27, 50 and 55) as predictors. These could classify resistant and susceptible female mosquitoes with a mean error rate of < 0.1. Similar RF strategies were also followed for the classification of samples according to Body Part (legs or remaining bodies).

2. Quantitation of chitin

Chitin determination in mosquito bodies was made according to Lehman and White (1975). Briefly, bodies (no legs) from both mosquito strains (susceptible and resistant) in three replicates of 8 female mosquitoes each, 3-5 days old, (48 legs each replicate) were mechanically homogenized and further processed for deacetylation of chitin to chitosan and determination of chitosan (i.e., glucosamine polymer). Samples were transferred to a 96-well microplate and absorbance was determined at 650nm in a plate reader (Molecular Devices, Spectra Max). Before chitin quantification, mosquito’s body weight was measured and used for normalization.

3. Quantitative real-time PCR (qPCR)

3-5 days old non-blood-fed female mosquito legs, both resistant and susceptible, were dissected and total RNA was isolated (N=3 pools of legs from 15 individuals, 90 legs totally), using the commercially available ARCTURUS® PicoPure® RNA Isolation Kit (Arcturus, Applied Biosystems, USA). The RNA extraction and cDNA synthesis protocols were performed according to the manufacturers’ instructions (Minotech 801-

1(10KU). Quantitative real-time PCR (qPCR) was used for the quantification of Chitin synthase1 (CHS1, accession number AGAP001748-RA) and Chitin synthase 2 (CHS2, accession number AGAP001205). The expression level was normalized against two validated reference genes Elongation factor Tu (Ef, accession no. AGAP005128) and 40S ribosomal protein (S7, accession no AGAP010592). Gene-specific primers are detailed in Table S4. Samples were run on a CFX Connect™ Real-Time PCR Detection System (#1855201, Bio-Rad). The qRT-PCR reaction was heated to 50°C for 15min, 95°C for 3min, followed by 40 cycles of 95°C for 10 s, 60°C for 45 s, and then a melting curve step (95°C for 10 s, 65°C for 5 s and 95°C for 0.5s). Results were analyzed by the Bio-Rad CFX Manager TM 3.1 software. Fold change was calculated from three biological replicates, three technical replicates and normalized against the housekeeping genes using the 2-ΔΔCT method (2). The statistical significance of gene expression fold change was calculated using the t-test (Excel and SPSS 18.0 Software). P-values lower than 0.05% were considered statistically significant (*).

Figure Legends

Figure S1: Proteomic analysis of the leg proteome, in insecticide resistant and susceptible *An gambiae* females.

A. COG-ONTOLOGY analysis of the leg-proteome (both resistant and susceptible mosquitoes). Functional categories enriched in the leg proteome identified by the Database for Annotation, Visualization, and Integrated Discovery (DAVID). Functional categories are sorted by their corrected P-value. *$P<0.1$ and **$P<0.05$. Fold Enrichment: the number of proteins found in the protein list with a certain GO term divided by the number of genes expected (based on the genome gene predictions of *Anopheles gambiae* str. PEST, with the particular GO term).

B. A pie chart of the 316 differentially regulated leg proteins: 156 proteins were up-, 160 were down-regulated, and the remaining 804 proteins were non-regulated in the resistant leg proteome, based on fold change criteria (see material and methods).

C. GO terms enrichment analysis of differentially expressed leg proteins and pathway enrichment analysis by g:Profiler. All terms in Molecular Function (MF) category and Biological process (BP) and pathways are sorted by their corrected p-value and the threshold of significance is at $P\leq 0.05$. **Up.** GO-MF of all up-regulated leg proteins (orange bars) revealed significantly enriched structural constituent of the cuticle and hydrogen ion transmembrane transporter activity molecular functions (corrected p-value= 5,04e-09 and 3,21e-02, respectively). **Middle.** GO-BP of all regulated proteins showed no enrichment in the set of the up-regulated proteins, but emerged small molecule metabolic process (corrected p-value= 1,58e-09), IMP metabolic process (corrected p-value= 7,24e-06) and nucleoside monophosphate biosynthetic process (corrected p-value= 1,43e-02) enriched in the down-regulated proteins. **Down.** Pathway enrichment analysis by KEGG of the up-regulated leg proteins (Orange bar) depicted Oxidative phosphorylation as primary pathway (corrected p-value= 1.16e-03). Pathway enrichment analysis by KEGG of the down-regulated proteins (black bars) revealed mainly Metabolic pathways enriched (Metabolic pathways, corrected p-value= 5,07e-05, Carbon metabolism, corrected p-value= 6,19e-03 and Tryptophan metabolism, corrected p-value= 3,72e-02).

Figure S2: **Differential expression of CPs in the resistant female legs**.

A. 37 Cuticular proteins and extra 4 proteins annotated as chitin binding, identified in total of 1120 leg proteins (Count: 41 proteins) (41/1120=3.6%). Out of them, 29 proteins were present in the set of the 156 up-regulated leg proteins (29/156= 18.6%). Enrichment ratio (5.1) was calculated as the ratio of 18.6% versus 3.6% (18.6%/3.6%=5.1).

B. Heat map of all cuticular proteins showing their regulation in the leg-proteome of *Anopheles* resistant mosquito legs (Fold Change expression resistant versus susceptible legs: RL/SL). Red color indicates increased abundance while green decreased abundance. Black indicates no significant fold change. Threshold fold change of differential expression >3.0 and <0.2 (fold change is normalized to median value).

Figure S3: Random Forest analysis of CHCs in the legs and bodies of resistant and susceptible female *An gambiae* mosquitoes.

A. Samples obtained from female legs and bodies were analyzed with a Random Forest Approach as described in Methods, using 35 peaks that passed the criteria for abundance (>0.1%). Peaks ranked for importance expressed as Mean Decrease of Gini Coefficient. Dashed grey line denotes threshold of 1.5 times the mean importance which was set in order to select the best predictors.

B. Samples obtained from female legs resistant and susceptible were used to predict susceptibility status with a Random Forest Approach as described in Methods. As in figure S1, only 35 peaks that passed the criteria for abundance (>0.1%) were incorporated in the model. Exactly as in figure S1, the best predictors were selected.

C. A characteristic GC-MS chromatograph of female resistant legs summarizing the major CHC species for the differentiation of legs from remaining bodies (gray triangles) and resistant from susceptible legs (green squares).

Figure S4: **Heat maps and hierarchical clustering** showing the relative amounts of CHCs, the best predictors in discriminating body parts (A) and resistant or susceptible legs (B). Red color indicates increased abundance while blue decreased abundance.

Figure S5: **Chitin content of resistant *An gambiae* female mosquito bodies.**

Chitin monomer quantitation (mgr of D-glucosamine/ mgr of mosquito bodies) in resistant (R: VK7) and susceptible (S: NG) female bodies (without legs). The bodies from eight female mosquitoes were analyzed and measurements were repeated three

times. Significance determined by *t*-test (non-significant) and the data are presented as means + SEM.

Figure S6: **Differential expression levels of chs1 in the legs by quantitative RT-PCR.**

csh1 was found 3.83 folds significantly up-regulated in the resistant leg compared to the susceptible ones. Data are presented as mean + SEM, n=3 biological replicates (*t*-test, *P<0.05).

Figures

Figure S1

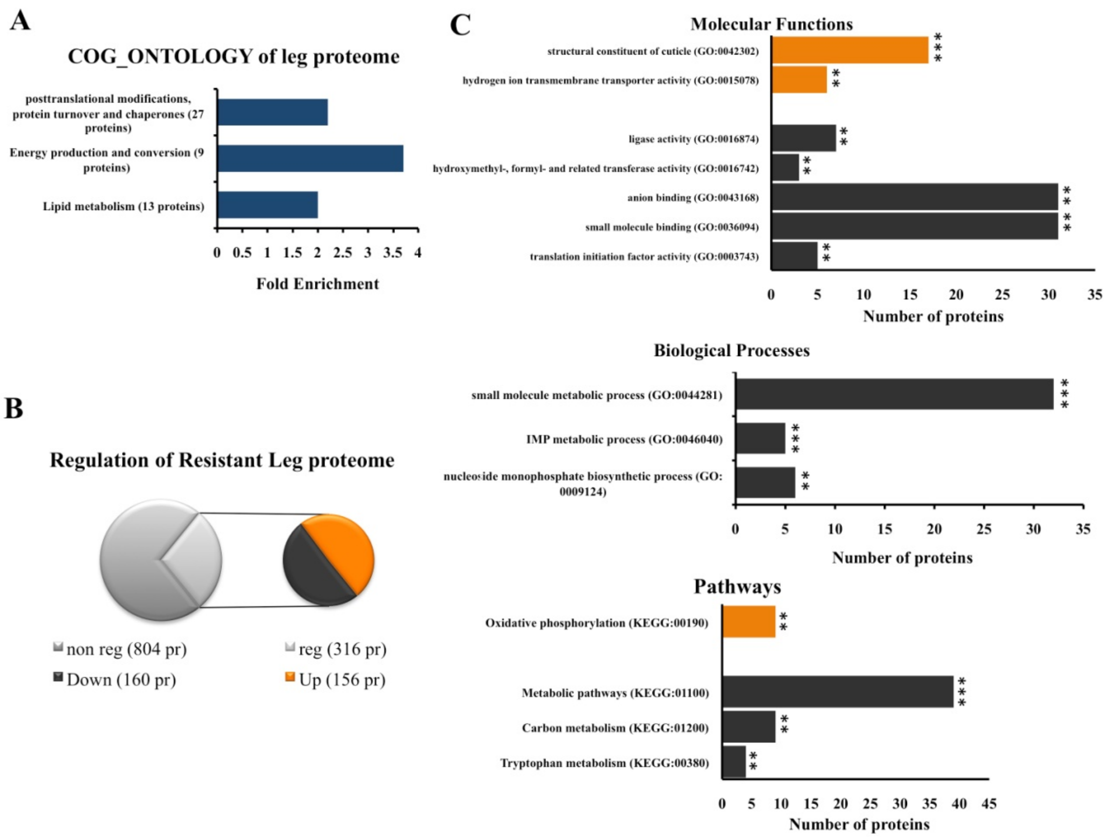

Figure S2

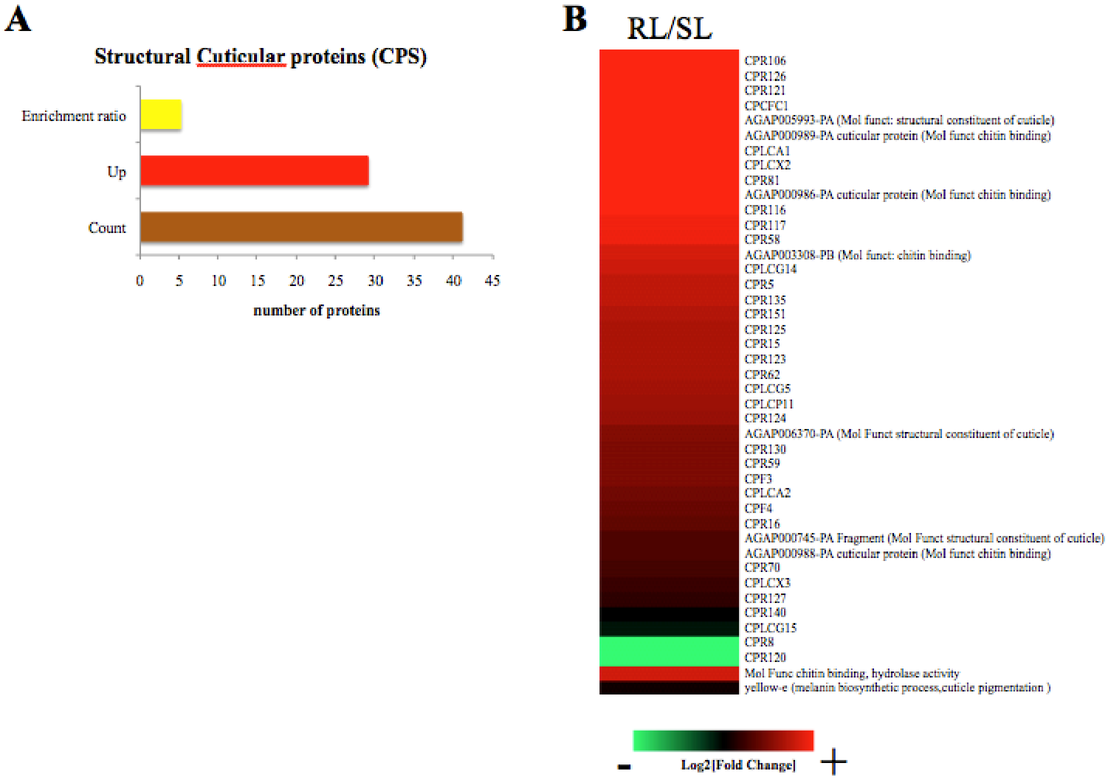
A
Structural Cuticular proteins (CPS)
Enrichment ratio
Up
Count
0
5
10
15
20
25
30
35
40
45
number of proteins
B
RL/SL
CPR106
CPR126
CPR121
CPCFC1
AGAP005993-PA (Mol funct: structural constituent of cuticle)
AGAP000989-PA cuticular protein (Mol funct chitin binding)
CPLCA1
CPLCX2
CPR81
AGAP000986-PA cuticular protein (Mol funct chitin binding)
CPR116
CPR117
CPR58
AGAP003308-PB (Mol funct: chitin binding)
CPLCG14
CPR5
CPR135
CPR151
CPR125
CPR15
CPR123
CPR62
CPLCG5
CPLCP11
CPR124
AGAP006370-PA (Mol Funct structural constituent of cuticle)
CPR130
CPR59
CPF3
CPLCA2
CPF4
CPR16
AGAP000745-PA Fragment (Mol Funct structural constituent of cuticle)
AGAP000988-PA cuticular protein (Mol funct chitin binding)
CPR70
CPLCX3
CPR127
CPR140
CPLCG15
CPR8
CPR120
Mol Func chitin binding, hydrolase activity
yellow-e (melanin biosynthetic process,cuticle pigmentation)
-
Log2[Fold Change]
+

Figure S3

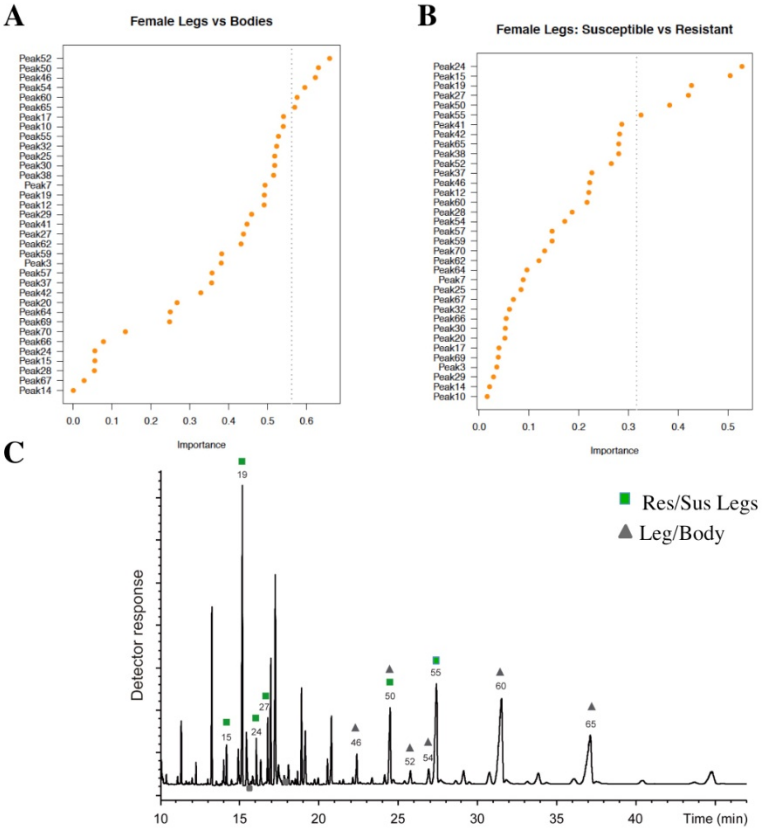
A
Female Legs vs Bodies
Peak52
Peak50
Peak46
Peak54
Peak60
Peak65
Peak17
Peak10
Peak55
Peak32
Peak25
Peak30
Peak38
Peak7
Peak19
Peak12
Peak29
Peak41
Peak27
Peak62
Peak59
Peak3
Peak57
Peak37
Peak42
Peak20
Peak64
Peak69
Peak70
Peak66
Peak24
Peak15
Peak28
Peak67
Peak14
0.0 0.1 0.2 0.3 0.4 0.5 0.6
Importance
B
Female Legs: Susceptible vs Resistant
Peak24
Peak15
Peak19
Peak27
Peak50
Peak55
Peak41
Peak42
Peak65
Peak38
Peak52
Peak37
Peak46
Peak12
Peak60
Peak28
Peak54
Peak57
Peak59
Peak70
Peak62
Peak64
Peak7
Peak25
Peak67
Peak32
Peak66
Peak30
Peak20
Peak17
Peak69
Peak3
Peak29
Peak14
Peak10
0.0 0.1 0.2 0.3 0.4 0.5
Importance
C
Res/Sus Legs
Leg/Body
Detector response
19
15
24
27
46
50
52
54
55
60
65
10 15 20 25 30 35 40 Time (min)

Figure S4

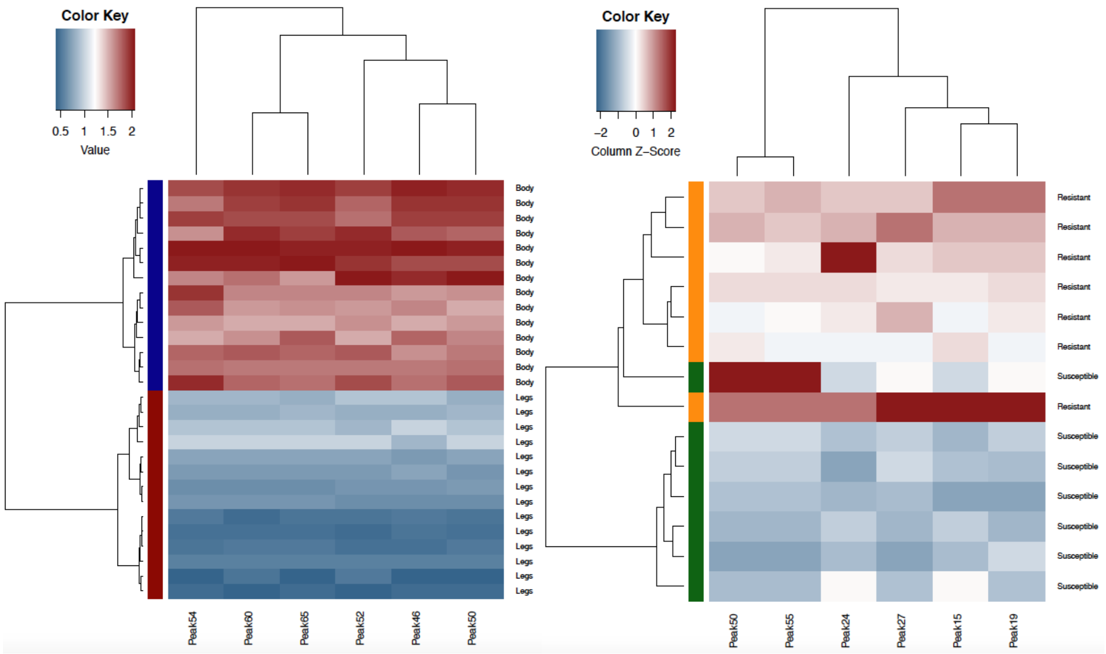
Color Key
0.5 1 1.5 2
Value
Body
Legs
Peak54
Peak60
Peak65
Peak52
Peak46
Peak50
Color Key
−2 0 1 2
Column Z−Score
Resistant
Susceptible
Peak50
Peak55
Peak24
Peak27
Peak15
Peak19

Figure S5

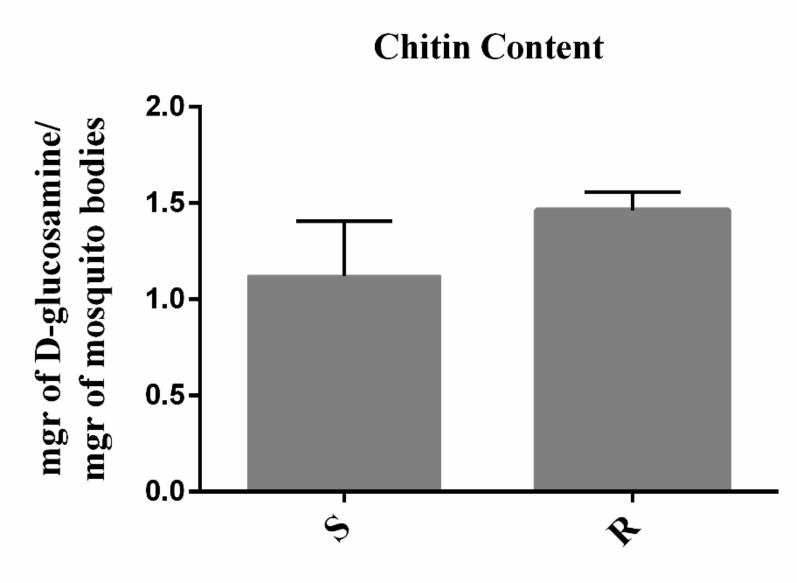
Chitin Content
mgr of D-glucosamine/
mgr of mosquito bodies
2.0
1.5
1.0
0.5
0.0
S
R

Figure S6

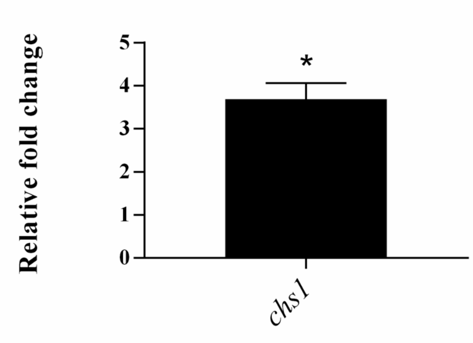

Tables

Table S1. **CHCs species that differentiate between legs and bodies**

CHC Species (peak number)
x-dimethyl C37 (46)
x, x-dimethyl C39 (50)
x, x-dimethyl C40 (52)
13-;19-;21-;15-;17-methyl C41 (54)
x, x-dimethyl C43 (65)
x, x-dimethyl C45

CHCs species are sorted by their elution time in the chromatograph (x: site of methyl group insertion). Full identification is available in Balabanidou *et al.*, 2016. Each peak contain a mixture of different isomers, with predominance of 13,x-; 11,x- and 15,x- components where the second methyl branching (x) is separated from the former by 3, 5, 7, or up to 2 carbons. CHCs from female mosquito body parts, both resistant and susceptible, were analyzed.

Table S2. CHCs species differentiated between legs from resistant and susceptible female *An gambiae* mosquitoes

CHC species (peak numbers)
nC28 (15)
nC29 (19)
nC30 (24)
nC31:1; 3-methyl C30 (27)
x, x-dimethyl C39 (50)
x,x-dimethyl C41 (55)

CHCs species are sorted by their elution time in the chromatograph (x: site of methyl group insertion). Full identification is available in Balabanidou *et al.*, 2016. CHCs from female mosquito legs (resistant versus susceptible) were analyzed.

Table S3. Differential expression levels of Detox-related proteins (Phase I and II), Antioxidants, Cuticular and Odorant Binding proteins in insecticide resistant mosquito legs compared to susceptible legs by label-free quantitative proteomics (LFQ).

Functional Annotation Cluster		Biological Variability		Fold Change (Norm. Median)	bb (*P*-value)
		Res Legs	Susc Legs		
**PHASE I**					
Cytochromes P450's					
**Accession number**	**Protein name**				
1. AGAP009246-PA	CYP4C27	low	medium	SL	0.02
2. AGAP012957- PA	CYP4D17	low	medium	SL	0.06
Epoxidehydrolases					
**Accession number**	**Protein name**				
1. AGAP011972-PA	Epoxide hydrolase	low	medium	SL	0.02
Carboxylesterases					
**Accession number**	**Protein name**				
1. AGAP005837-PA	carboxylesterase	medium	medium	0.05	0.1
2. AGAP006227-PA	alpha-esterase	low	medium	SL	0.06
**PHASE II**					
Glutathione S-transferases					
**Accession number**	**Protein name**				
1. AGAP009197-PA	GSTe3	medium	low	RL	0.05
2. AGAP000165-PA	GSTmic1	medium	medium	3.17	0.2
3. AGAP005749-PA	GSTO1	medium	medium	0.08	0.009
**ANTIOXIDANT ENZYMES**					
Superoxide dismutases					
**Accession number**	**Protein name**				
1. AGAP010347-PB	CUSOD3	medium	medium	14.83	0.065
**CUTICULAR PROTEINS (CPs)**					
**Accession number**	**Protein name**				
1. AGAP000345-PA	CPR126	medium	medium	65	0.03
2. AGAP003383-PA	CPR121	high	medium	14.16	0.01

3. AGAP003379-PA	CPR117	high	high	8.33	0.05
4. AGAP001668-PA	CPR5	medium	medium	5.33	0.11
5. AGAP006261-PA	CPR135	medium	medium	5.33	0.18
6. AGAP009870-PA	CPR151	medium	medium	4.83	0.15
7. AGAP000820-PA	CPR125	high	high	4.5	0.12
8. AGAP007042-PA	CPR62	medium	medium	4.33	0.19
9. AGAP006829-PA	CPR59	high	high	3	0.04
10. AGAP000047-PA	CPR130	high	high	3	0.05
11. AGAP003382-PA	CPR120	low	medium	SL	0.08
**ODORANT BINDING PROTEINS**					
**Accession number**	**Protein name**				
1. AGAP001189-PB	OBP10	medium	medium	6.33	0.08
2. AGAP011368-PA	OBP57	medium	medium	3.67	0.01
3. AGAP002905-PA	OBP13	medium	medium	0.17	0.06
4. AGAP008281-PA	D7-related 4 protein	medium	medium	0.07	0.09
5. AGAP008282-PA	D7-related 2 protein	medium	medium	0.05	0.006
6. AGAP008279-PA	D7 long form salivary protein	low	medium	SL	0.08
7. AGAP006080-PA	OBP5470	medium	medium	SL	0.1

Biological variability indicates standard deviation between biological replicates (low: 10^-4, medium: <10^-1 and >10^-4, high: >10^-1).

bb (*P*-value): Beta-bionomial testing using the ibb library in R, p-values provided. Values below a certain value (0.1) were considered significantly differentially expressed.

Proteins presented are considered significantly differentially regulated based on two criteria: I) Threshold Fold Change (Normalized to Median)>3.0 for up-regulation and <0.2 for down-regulation and II) bb (*P*-value) <0.1.

SL: Indicated proteins identified only in susceptible legs.

RL: Indicated proteins identified only in resistant legs.

Table S4. RT-PCR primers used in this study.

Gene	Description	Accession number	Primer name	Sequence (5' to 3')	Transcipt length (bp)
CHS1	Chitin synthase 1	AGAP001748	CHS1F	GCTCTCATAGACAAGCACGCTG	190
			CHS1R	TGCCTTTTCGAGGTTCTGGA	
CHS2	Chitin synthase 2	AGAP001205	CHS2F	GAAGGCACTGGAAATGAAATTAACC	163
			CHS2R	TTTTCACCGTCTGGATCGGG	
EF	Elongation factor Tu	AGAP005128	EFF	GGCAAGAGGCATAACGATCAATGCG	130
			EFR	GTCCATCTGCGACGCTCCGG	
S7	40S ribosomal protein	AGAP010592	S7F	AGAACCAGCAGACCACCATC	149
			S7R	GCTGCAAACTTCGGCTATTC	

References

1. Aivaliotis M, Haase W, Karas M, Tsiotis G. Proteomic analysis of chlorosome-depleted membranes of the green sulfur bacterium Chlorobium tepidum. Proteomics. 2006;6(1):217-32. Epub 2005/11/19.
2. Schmittgen TD, Livak KJ. Analyzing real-time PCR data by the comparative C(T) method. Nat Protoc. 2008;3(6):1101-8.
